# Toggling between gamma-frequency activity and suppression of cell assemblies

**DOI:** 10.3389/fncom.2013.00033

**Published:** 2013-04-16

**Authors:** Christoph Börgers, Bryan Walker

**Affiliations:** Department of Mathematics, Tufts UniversityMedford, MA, USA

**Keywords:** gamma oscillation, feedback inhibtion, cell assembly, attentional selection, type 2 neuron

## Abstract

Gamma (30–80 Hz) rhythms in hippocampus and neocortex resulting from the interaction of excitatory and inhibitory cells (E- and I-cells), called Pyramidal-Interneuronal Network Gamma (PING), require that the I-cells respond to the E-cells, but don't fire on their own. In idealized models, there is a sharp boundary between a parameter regime where the I-cells have weak-enough drive for PING, and one where they have so much drive that they fire without being prompted by the E-cells. In the latter regime, they often de-synchronize and suppress the E-cells; the boundary was therefore called the “suppression boundary” by Börgers and Kopell (2005). The model I-cells used in the earlier work by Börgers and Kopell have a “type 1” phase response, i.e., excitatory input always advances them. However, fast-spiking inhibitory basket cells often have a “type 2” phase response: Excitatory input arriving soon after they fire delays them. We study the effect of the phase response type on the suppression transition, under the additional assumption that the I-cells are kept synchronous by gap junctions. When many E-cells participate on a given cycle, the resulting excitation advances the I-cells on the next cycle if their phase response is of type 1, and this can result in suppression of more E-cells on the next cycle. Therefore, strong E-cell spike volleys tend to be followed by weaker ones, and vice versa. This often results in erratic fluctuations in the strengths of the E-cell spike volleys. When the phase response of the I-cells is of type 2, the opposite happens: strong E-cell spike volleys delay the inhibition on the next cycle, therefore tend to be followed by yet stronger ones. The strengths of the E-cell spike volleys don't oscillate, and there is a nearly abrupt transition from PING to ING (a rhythm involving I-cells only).

## 1. Introduction

Gamma-frequency (30–80 Hz) oscillations in hippocampus and neocortex are known to result, in many instances, from the interaction of excitatory pyramidal cells (E-cells) and fast-spiking inhibitory interneurons (I-cells) (Whittington et al., 2000; Börgers and Kopell, 2003; Bartos et al., 2007; Traub and Whittington, 2010). Rhythms arising in this way are called Pyramidal-Interneuronal Network Gamma (PING) rhythms. The PING mechanism requires that the I-cells respond to the E-cells, but do not fire on their own; thus the drive to the I-cells must be sufficiently weak, in comparison with the drive to the E-cells. In idealized model networks, there can be a sharp boundary in parameter space between a regime in which the I-cells have weak-enough drive for PING, and a regime in which they have so much drive that they fire without being prompted by the E-cells. In the latter regime, they often de-synchronize, and suppress the E-cells altogether; the boundary in parameter space was therefore called the “suppression boundary” in Börgers and Kopell (2005). [The loss of synchrony among the I-cells is the result of heterogeneity in drives (White et al., 1998), and would not be expected in a homogeneous network (Achuthan and Canavier, 2009)]. However, the transition from PING to suppression is truly discontinuous only under very idealized circumstances. We therefore replace the term “suppression boundary” by “suppression transition” in this paper. Even in networks with heterogeneous neuronal properties, this transition can be narrow (Börgers et al., 2008). Thus, a small amount of modulation of the excitability of the neurons can result in crossing from the PING regime to the suppression regime or vice versa, and therefore cause a dramatic change in network dynamics. In Börgers et al. (2005), it was explained how this mechanism could be exploited in attentional processing, turning on or off the processing of certain stimuli.

There was no gap-junctional coupling among I-cells in Börgers and Kopell (2005) and Börgers et al. (2005), even though such coupling is known to be present among fast-spiking interneurons in neocortex (Galarreta and Hestrin, 2002) and hippocampus (Fukuda and Kosaka, 2000). Furthermore, the model I-cells used in Börgers and Kopell (2005) and Börgers et al. (2005) have a type 1 phase response, i.e., excitatory input always advances them. However, fast-spiking inhibitory basket cells often have a type 2 phase response: they are delayed by excitatory input arriving soon after they fire (Tateno and Robinson, 2007, Figure 5). We study the effect on the suppression transition of introducing I-cells with type 2 phase response, and coupling them with gap junctions strong enough to keep them synchronous (Kopell and Ermentrout, 2004; Ostojic et al., 2009).

The type of the phase response is not the only notion of neuronal type in mathematical neuroscience. The bifurcation from rest to spiking is said to be of type 1 if it is a saddle-node bifurcation on an invariant circle, and of type 2 if it is a Hopf bifurcation (Rinzel and Ermentrout, 1998). The frequency-current (*f* - *I*) relation is said to be of type 1 if it has no discontinuity, i.e., if arbitrarily slow spiking is possible for drives sufficiently close to (but above) threshold, and of type 2 if it has a discontinuity at spike onset. [This last notion of neuronal type was described by Hodgkin (1948)]. The three kinds of neuronal type often coincide. For instance, the classical Hodgkin–Huxley model is of type 2 according to all three definitions, and for each of the three model neurons used in this paper, the three notions of type coincide. However, there is reason to be cautious about identifying the types of bifurcations, phase response curves, and *f* - *I* relations: Ermentrout et al. (2012) recently gave an example showing that a type 1 bifurcation can be associated with a type 2 phase response. What matters to us in this paper is the type of the phase response. When we call a model neuron “of type 1,” we mean that weak excitatory inputs always accelerate it. When we call it “of type 2,” we mean that weak excitatory inputs arriving early in the cycle hold it back.

If type 2 I-cells are introduced in the models of Börgers and Kopell (2005) and Börgers et al. (2005), but without gap-junctional coupling, or if the type 1 I-cells are kept, but coupled by synchronizing gap junctions, we find that the suppression transition becomes considerably less tight. However, a sharp suppression transition is restored when I-cells of type 2 and synchronizing gap-junctional coupling among them are introduced at the same time; crossing it causes a nearly abrupt transition from PING to ING (Whittington et al., 2000), i.e., to a gamma rhythm involving the rhythmic firing of I-cells only, with the E-cells suppressed. We give an analysis explaining why in the presence of synchronizing gap junctions among the I-cells, the suppression transition is much tighter with I-cells of type 2 than with I-cells of type 1.

In summary, the idea that the suppression transition may play a central role in attentional processing remains intact when the I-cells are of type 2, connected by gap junctions.

## 2. Models

### 2.1. A variation of the erisir interneuron model

Erisir et al. (1999) proposed a model of inhibitory interneurons in mouse somatosensory cortex. We use it here because it is the simplest Hodgkin–Huxley-like interneuron model of type 2 that we know of. Because several variants of the Erisir model have appeared in the literature, and because we use a variant slightly different from any of those in the literature, we will state our equations here. The form of the differential equations is

(1) $$C\frac{dv}{dt}=g_{Na}m_{\infty }{\left(v\right)}^{3}h(v_{Na}-v)+g_{K}n^{2}(v_{K}-v)+g_{L}(v_{L}-v)+I,$$

(2) $$\frac{dx}{dt}=\frac{x_{\infty }(v)-x}{\tau_{x}(v)},\;x=h,n.$$

Deviating from Erisir et al. (1999), we take the activation variable *m* of the sodium current to be a direct function of *v*. Following Erisir et al. (1999), the second power of *n* appears in the delayed rectifier potassium current, even though in the original Hodgkin–Huxley model (Hodgkin and Huxley, 1952) and almost all similar models, the fourth power appears there. The original model of Erisir et al. (1999) also included a weak, slow, depolarization-induced potassium current, which plays no role in our discussion, and will be omitted here.

The letters *C*, *v*, *t*, *g*, and *I* in Equations (1) and (2) denote capacitance density, voltage (membrane potential), time, conductance density, and current density, respectively, measured in μF/cm^2^, mV, ms, mS/cm^2^, and μA/cm^2^; we will usually omit units from here on. The reversal potentials are, following (Erisir et al., 1999), *v*_*Na*_ = 60, *v*_*K*_ = −90, *v*_*L*_ = −70. Erisir et al. specified conductances and currents; to translate to conductance and current *densities*, we assume, following Erisir et al., that the neuron is a sphere of radius 8 μm. The parameter choices of (Erisir et al., 1999) then become, using the units specified above and rounding to three significant digits, *C* = 1, *g*_*Na*_ = 112, *g*_*K*_ = 224, and *g*_*L*_ = 1.24. Gouwens et al. (2010) reduced the leak conductance, using a value which translates into a conductance density of approximately 0.5 mS/cm^2^; this is the value that we use here. The lowest possible firing frequency of the Erisir neuron with *g*_*L*_ = 1.24 is quite high, about 65 Hz; with *g*_*L*_ = 0.5, it is significantly lower, approximately 37 Hz. The gating variables *m*, *h*, and *n* are non-dimensional quantities varying between 0 and 1. The equations for *x*_∞_ (*x* = *m*, *h*, *n*) and τ_*x*_ (*x* = *h*, *n*) are

$$\begin{array}{l} \;x_{\infty }=\frac{\alpha_{x}}{\alpha_{x}+\beta_{x}},\;x=m,\;h,\;n,\\ \;\tau_{x}=\frac{1}{\alpha_{x}+\beta_{x}},\;x=h,\;n,\\ \alpha_{m}(v)=40(75.5-v)/(\exp((75.5-v)/13.5)-1),\\ \beta_{m}(v)=1.2262/\exp(v/42.248),\\ \alpha_{h}(v)=0.0035/\exp(v/24.186)),\\ \beta_{h}(v)=-0.017(v+51.25)/(\exp(-(v+51.25)/5.2-1),\\ \alpha_{n}(v)=(95-v)/(\exp((95-v)/11.8)-1),\\ \beta_{n}(v)=0.025/\exp(v/22.222). \end{array}$$

We have corrected a typographical error in the formula for α_*m*_ in Erisir et al. (1999) [pointed out by (Gouwens et al., 2010)], and made a slight correction in the formula for β_*h*_: Erisir et al. wrote 0.8712 + 0.017*v* instead of 0.017(*v* + 51.25). Up to rounding, these two expressions are equal, but if one writes 0.8712 + 0.017*v*, then β_*h*_ has a singularity, since the denominator vanishes at *v* = −51.25, whereas the numerator vanishes at *v* = −0.8712/0.017 ≈ 51.247; we have found that this can in fact have adverse effects during simulations.

We define the “firing times” of the neurons as times at which *v* = −20 and *dv*/*dt* < 0. If the firing period is *T* > 0, the frequency is *f* = 1000/*T*. The factor of 1000 arises because we measure time in ms, but frequencies not in reciprocal ms, but in reciprocal s, namely, in Hz.

**Figure 1:**
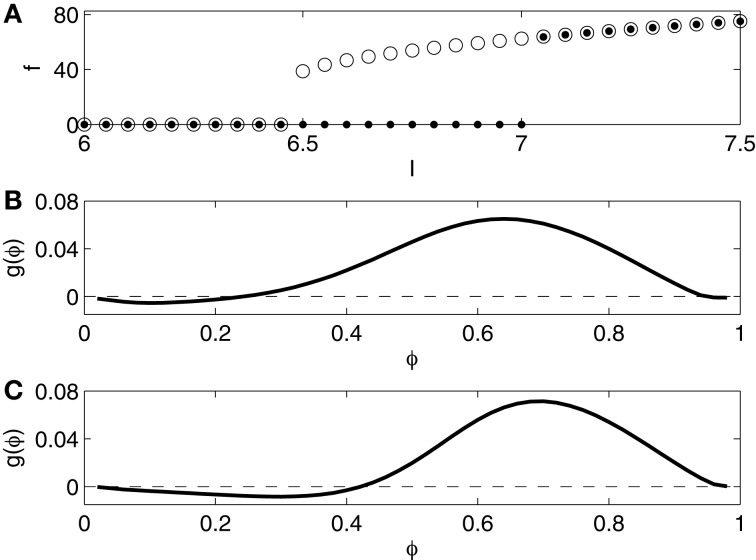
**(A)** *f* - *I* relation for the Erisir interneuron. Dots show *f* as a function of *I* as *I* is slowly increased. Open circles show *f* as a function of *I* as *I* is slowly decreased. **(B)** Phase response of the Erisir interneuron (*I* = 7.2) to an instantaneous increase in the membrane potential by 1 mV. **(C)** Phase response of the Erisir interneuron (*I* = 7.2) with an inhibitory autapse (modeled as described in Section 2.4, with maximal conductance equal to 0.2) to an instantaneous increase in the membrane potential by 1 mV.

We compute the frequency-current (*f* - *I*) relation of the Erisir model neuron as follows. We begin with a simulation for *I* = 6, starting at (*v*, *h*, *n*) = (−20, 1, 0). The computed trajectory converges to a fixed point which appears to be globally attracting. We then raise *I*, in steps of 0.05, from 6.0 to 7.5, starting each new simulation at the point in phase space at which the previous simulation ended. As soon as *I* rises above 7.0 (approximately), periodic spiking begins, at an onset frequency of approximately 60 Hz. The dots in Figure 1A indicate the spiking frequency *f* as a function of *I*. We then lower *I*, in steps of 0.05, from 7.5 to 6, again starting each new simulation at the point in phase space at which the previous simulation ended. Periodic spiking continues as *I* falls below 7, and ceases only when *I* falls below 6.5. The circles in Figure 1A indicate *f* as a function of *I*, as *I* is gradually lowered. For *I* approximately between 6.5 and 7, there is bi-stability: both rest and periodic spiking are possible and stable in this range.

The *f* - *I*-relation shown in Figure 1A is typical of a subcritical Hopf bifurcation (Strogatz, 1994). Specifically, the figure suggests that the resting state loses its stability in a subcritical Hopf bifurcation as *I* rises above approximately 7, and the stable limit cycle corresponding to periodic spiking is annihilated, likely in a saddle-node bifurcation of cycles (Strogatz, 1994), as *I* falls below approximately 6.5.

We also compute a phase response curve for the Erisir interneuron, defined as follows. Assume that *I* is large enough to allow periodic firing (above 6.5, approximately). Denote the firing period by *T*. Suppose that (*v*, *h*, *n*) = (−20, *h*_0_, *n*_0_) is the uniquely determined point on the limit cycle with *v* = −20 and *dv*/*dt* < 0. At time *t* = 0, we start a simulation at this point. At time φ*T*, with 0 < φ < 1, we abruptly increase *v* by 1 mV; this corresponds to an instantaneous charge injection at time φ*T*. Denote by $\tilde{T}$ the next time when *v* = −20, *dv*/*dt* < 0. The phase advance produced by the input is

$$g(\varphi )=\frac{T-\tilde{T}}{T}.$$

Figure 1B shows the phase response curve, that is, the graph of the function *g*, for *I* = 7.2. It is of type 2: Excitatory input received early in the cycle delays the next spike instead of advancing it. Since in our network simulations, the I-cells inhibit each other, we also compute the phase response curve for the Erisir interneuron with an inhibitory autapse, modeled as described in Section 2.4, with maximal conductance equal to 0.2. The self-inhibition makes the type 2 character of the phase response more pronounced; see Figure 1C.

**Figure 2:**
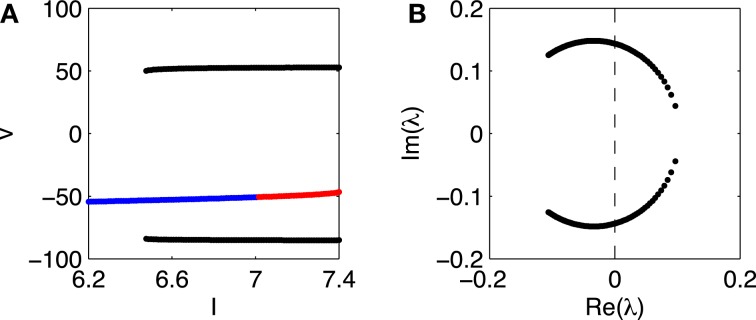
**(A)** Membrane potential *v* of fixed point of Erisir interneuron, as a function of *I* (blue and red indicate stability and instability, respectively), and maximum and minimum membrane potentials along the stable limit cycle (black). **(B)** Non-real eigenvalues of Jacobian at fixed point, for values of *I* as in panel **(A)**. The crossing of the imaginary axis from the left half plane to the right occurs for the critical value of *I* at which the fixed point in A loses its stability.

Figure 2 presents a closer look at the transition from rest to firing in the Erisir interneuron, and in particular provides strong evidence for a subcritical Hopf bifurcation. The figure shows the range 6.2 ≤ *I* ≤ 7.4. For *I* = 6.2, there is a single stable fixed point. Figure 2A tracks this fixed point as *I* rises.^1^ Figure 2A shows the membrane potential at the fixed point, as a function of *I*, with blue indicating stability, and red instability. At a value of *I* near 6.5, a stable limit cycle arises; Figure 2A indicates the maximum and minimum membrane potentials along the limit cycle in black. The fixed point becomes unstable at a value of *I* very slightly above 7.

To confirm that the fixed point loses its stability in a Hopf bifurcation, we plot, in Figure 2B, the non-real eigenvalues of the Jacobi matrix at the fixed point, for the range of drives *I* in Figure 2A. There is indeed a complex-conjugate pair of eigenvalues that crosses the imaginary axis. The parameter value at which the crossing occurs is ≈ 7.03.

### 2.2. The wang–Buzsáki (WB) interneuron model

**Figure 3:**
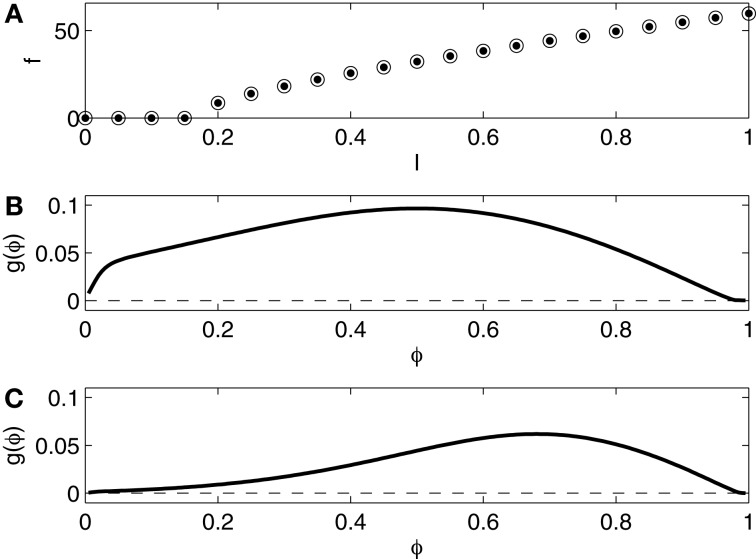
**(A)** *f* - *I* relation for the Wang–Buzsáki (WB) interneuron. Dots show *f* as a function of *I* as *I* is slowly increased. Open circles show *f* as a function of *I* as *I* is slowly decreased. The dots and the circles coincide for this model. **(B)** Phase response of the WB interneuron (*I* = 1.0) to an instantaneous increase in the membrane potential by 1 mV. **(C)** Phase response of the self-inhibiting WB interneuron (*I* = 1.0, maximal inhibitory conductance = 0.2) to an instantaneous increase in the membrane potential by 1 mV.

We compare networks in which the I-cells are the Erisir neurons presented in the previous section with networks in which they are Wang–Buzsáki (WB) neurons (Wang and Buzsáki, 1996), modeling fast-firing interneurons in rat hippocampus. We use the equations without any change from Wang and Buzsáki (1996). Figure 3 shows the *f* - *I* relation and the phase response curve, without self-inhibition (panel **B**) and with self-inhibition (panel **C**). The *f* - *I* relation now indicates no region of bi-stability, and is of type 1, with a square-root-like appearance. The phase response is of type 1 as well, positive throughout.

### 2.3. Reduced traub-miles (RTM) model of pyramidal neurons

**Figure 4:**
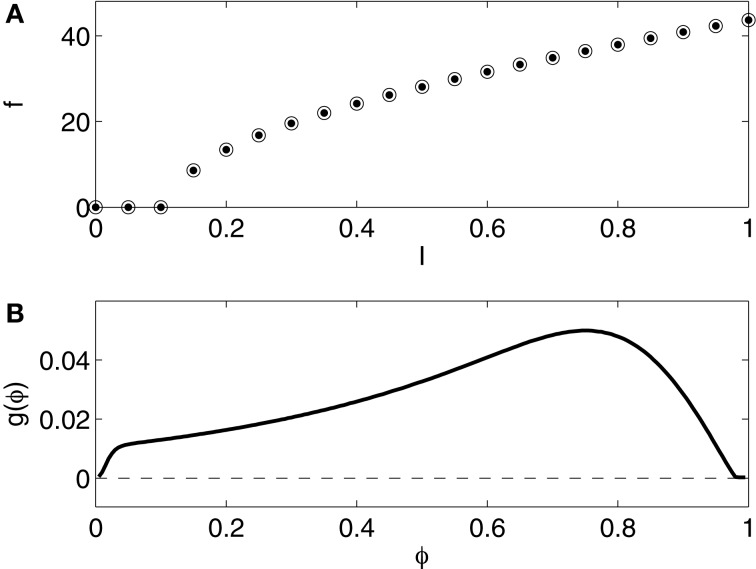
**(A)** *f* - *I* relation for the reduced Traub-Miles (RTM) rneuron. Dots show *f* as a function of *I* as *I* is slowly increased. Open circles show *f* as a function of *I* as *I* is slowly decreased. The dots and the circles coincide for this model. **(B)** Phase response of the RTM neuron (*I* = 1.0) to an instantaneous increase in the membrane potential by 1 mV.

The pyramidal cell model used in this article is that of Kopell et al. (2010). We refer to Kopell et al. (2010, Appendix 1) for the details. The model is a slight variation of that of Olufsen et al. (2003), which in turn is a slight variation of that of Ermentrout and Kopell (1998), a one-compartment reduction of a model of a rat hippocampal pyramidal neuron due to Traub and Miles (1991). Figure 4 shows the *f* - *I* relation and the phase response curve. The *f* - *I* relation and the phase response curve are of type 1.

### 2.4. Networks

We use the network model described in Kopell et al. (2010, Appendix 1). For clarity and completeness, we briefly recapitulate this model here, and we state the specific parameters used in our simulations.

**Figure 5:**
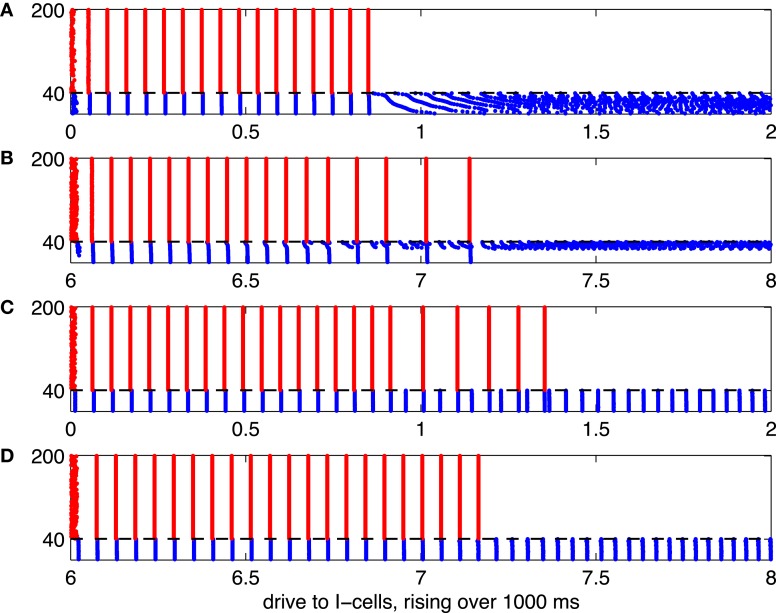
**Illustration of the suppression transition.** Red dots indicate spikes of E-cells, and blue dots indicate spikes of I-cells. The mean drive, *I*_*I*_, to the I-cells rises linearly, from 0 to 2 in panels **(A)** and **(C)**, and from 6 to 8 in panels **(B)** and **(D)**. The horizontal axis indicates *I*_*I*_ (or, equivalently, time in units of 500 ms). **(A)** I-cells are WB neurons, and there are no gap junctions. **(B)** I-cells are Erisir neurons, and there are no gap junctions. **(C)** I-cells are WB neurons, with gap junctions. **(D)** I-cells are Erisir neurons, with gap junctions.

**Figure 6:**
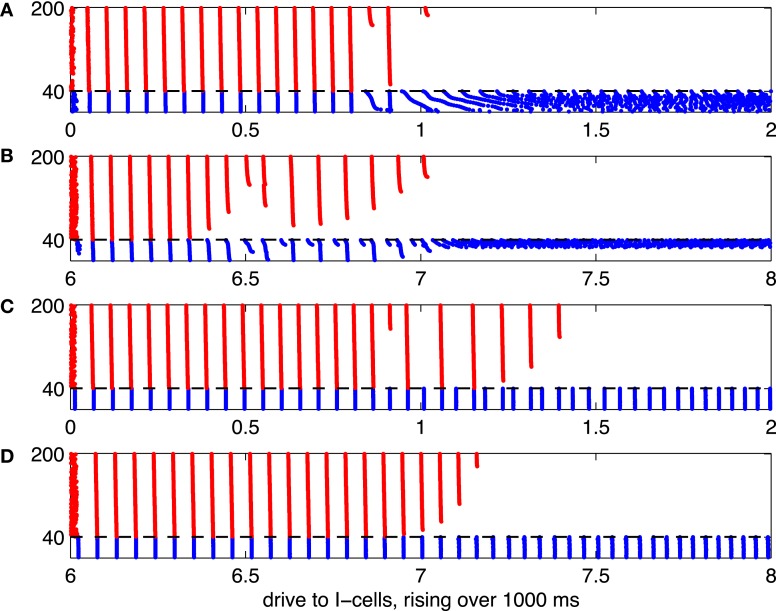
**As Figure 5, but with heterogeneous drive to the E-cells, rising with neuronal index from 1.85 to 2.15**.

**Figure 7:**
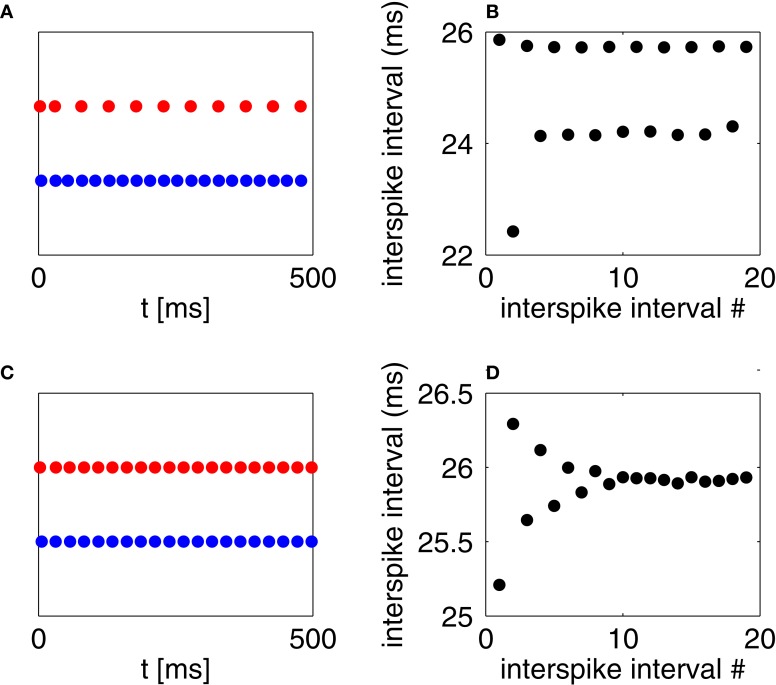
**(A)** Spikes of an RTM neuron (red) and a WB neuron (blue), with *I*_*E*_ = 2 and *I*_*I*_ = 1. **(B)** Inter-spike intervals of the I-cell in panel **(A)**, alternating between short ones (following a spike of the E-cell) and long ones (following suppression of the E-cell). **(C)** Spikes of an RTM neuron (red) and an Erisir neuron (blue), with *I*_*E*_ = 2 and *I*_*I*_ = 7.27. The E-cell would be entirely suppressed if *I*_*I*_ were 7.28. **(D)** Inter-spike intervals of the I-cell in panel **(C)**.

We denote by *N*_*E*_ the number of E-cells, and by *N*_*I*_ the number of I-cells. For our larger networks (Figures 5, 6, and 9), *N*_*E*_ = 160 and *N*_*I*_ = 40. We also report on numerical experiments with networks of one E- and one I-cell (Figure 7). The drive to each cell of the network is constant in time. We denote the drive to the *i*-th E-cell by *I*_*E*, *i*_, and the drive to the *j*-the I-cell by *I*_*I*, *j*_. The values of these drives will be varied; see Results.

Each synapse is characterized by a synaptic gating variable *s* associated with the presynaptic neuron, 0 ≤ *s* ≤ 1, with

$$\frac{ds}{dt}=\rho (v)\frac{1-s}{\tau_{R}}-\frac{s}{\tau_{D}},$$

where ρ denotes a smoothed Heaviside function, ρ(*v*) = (1 + tanh(*v*/4))/2, *v* denotes the presynaptic membrane potential, and τ_*R*_ and τ_*D*_ are the rise and decay time constants, respectively. To model the synaptic input from neuron *i* to neuron *j*, we add to the right-hand side of the equation governing the membrane potential *v*_*j*_ of neuron *j* a term of the form *g*_*ij*_*s*_*i*_(*t*) (*v*_*rev*_ − *v*_*j*_) where *g*_*ij*_ denotes the maximal conductance associated with the synapse, *s*_*i*_ denotes the gating variable associated with neuron *i*, and *v*_*rev*_ denotes the synaptic reversal potential. For excitatory, AMPA-receptor-mediated synapses, we use τ_*R*_ = 0.1, τ_*D*_ = 3, and *v*_*rev*_ = 0; for inhibitory, GABA_A_-receptor-mediated synapses, τ_*R*_ = 0.3, τ_*D*_ = 9, and *v*_*rev*_ = −80.

In comparison with many of the values reported in the literature, our choice of *v*_*rev*_ is low. Hyperpolarizing reversal potentials of GABA_A_-receptor-mediated inhibition have been reported, for instance, in Wang and Buzsáki (1996), Connors et al. (1988, Table 1), Sanchez-Vives and McCormick (1997), and Traub et al. (1996). Higher (sometimes much higher) reversal potentials have been reported by others; see for instance McCormick (1989); Vida et al. (2006); Bartos et al. (2007); Gouwens et al. (2010), but also Bregestovski and Bernard (2012). We have not systematically investigated the effect of a higher reversal potential on our conclusions. Powerful inhibition often seems to be needed for an abrupt suppression transition. However, inhibition can be sufficiently powerful for several reasons: low reversal potential, strong inhibitory conductance, or relatively low excitability of the post-synaptic cells.

**Figure 8:**
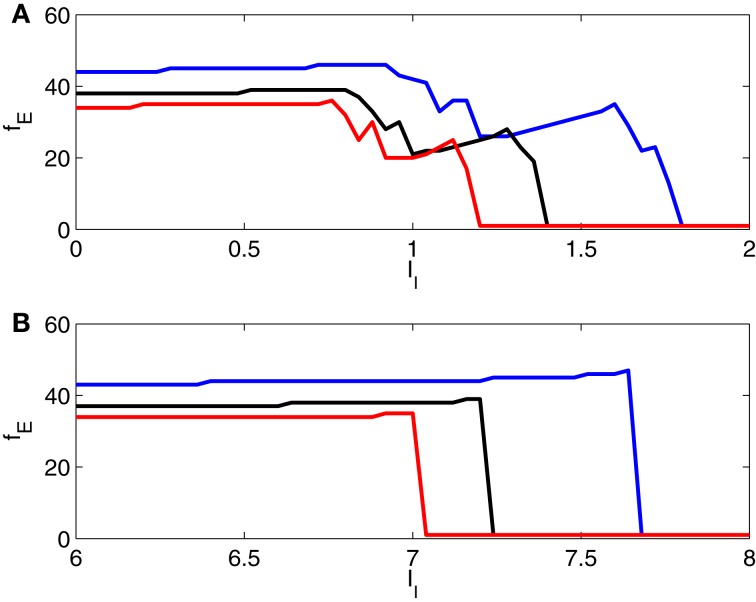
**Frequency of E-cell in a two-cell network as a function of drive to the I-cell, for *g*_*IE*_ = 0.5 (blue), 0.8 (black), and 1.1 (red).** The I-cell is either a WB neuron (panel **A**), or an Erisir neuron (panel **B**). The transition from firing to suppression of the E-cell is much cleaner and more abrupt with the Erisir neuron.

Connectivity is all-to-all in our networks. (Sparse, random connectivity would not yield substantially different results. The randomness would add heterogeneity, with effects similar to those of heterogeneity in external drives.) The value of *g*_*ij*_ depends only on the *types* (E or I) of neurons *i* and *j*. For instance, we denote by *g*_*EI*_ the value of *g*_*ij*_ when the *i*-th neuron is an E-cell, and the *j*-th neuron is an I-cell. Parameters *g*_*IE*_, *g*_*II*_, and *g*_*EE*_ are defined similarly. We scale these parameters with network size: *g*_*EI*_ = ĝ_*EI*_/*N*_*E*_, *g*_*IE*_ = ĝ_*IE*_/*N*_*I*_, *g*_*II*_ = ĝ_*II*_/*N*_*I*_, *g*_*EE*_ = ĝ_*EE*_/*N*_*E*_. We choose parameters similar to those in Börgers and Kopell (2005): ĝ_*EI*_ is so strong that a population spike volley of the E-cells promptly triggers one of the I-cells (but not much stronger), and ĝ_*IE*_ is significantly stronger: ĝ_*EI*_ = 0.2, ĝ_*IE*_ = 0.8, ĝ_*II*_ = 0.2, ĝ_*EE*_ = 0. The value of ĝ_*IE*_ is varied in Figure 8.

In some of our networks, the I-cells are also gap-junctionally coupled. The *i*-th and *j*-th I-cells are gap-junctionally connected with probability 1/5. If *v*_*i*_ and *v*_*j*_ are the membrane potentials of the two cells, we add to the right-hand side of the equation for *v*_*i*_ the term *g*_*gap*_(*v*_*j*_ − *v*_*i*_), and to the right-hand side of the equation for *v*_*j*_ the term *g*_*gap*_(*v*_*i*_ − *v*_*j*_), with *g*_*gap*_ = 0.8; this ensures that the I-cells remain synchronous when there are gap junctions.

Each figure in this paper was generated by a stand-alone Matlab code, available from the first author upon request.

## 3. Results

### 3.1. Simulation results for large networks

Figure 5 shows spike rastergrams resulting from simulations in which the mean drive to the I-cells, *I*_*I*_, increases linearly with time. The drive to the I-cells is heterogeneous, here and in all of our large network simulations; actual drive to the *j*-th I-cell is

$$I_{I,j}=\left(0.85+\frac{j-1/2}{40}\times 0.30\right)I_{I},\;1\leq j\leq 40.$$

The simulations are 1000 ms long, and *I*_*I*_ varies from 0 to 2 for WB neurons, and from 6 to 8 for Erisir neurons. The horizontal axis shows *I*_*I*_. Panel **(A)** of the figure illustrates the “suppression boundary” as described in Börgers and Kopell (2005). The I-cells are WB neurons here [in Börgers and Kopell (2005), they were theta neurons, which are also of type 1], and there is [as in Börgers and Kopell (2005)] no gap-junctional coupling among them. The abrupt cessation of gamma oscillations and suppression of the E-cells when the mean drive to the I-cells exceeds (approximately) 0.9 indicates the crossing of the suppression boundary.

Panels **(B)** through **(D)** of Figure 5 illustrate what happens when the WB interneurons are replaced by Erisir interneurons, or gap-junctional coupling among I-cells is introduced, or both. When only one of those two changes is made, the suppression transition broadens considerably, with a fairly large intermediate regime of cycle-skipping emerging (panels **B** and **C**). When both changes are made at the same time, however, one returns to an abrupt suppression transition (panel **D**). In the presence of gap junctions (panels **C** and **D** of Figure 5), the I-cells do not de-synchronize after they suppress the E-cells. The gap junctions together with the I→I-synapses synchronize the I-cells in spite of the fact that the external drive to the I-cells is heterogeneous (Kopell and Ermentrout, 2004).

In Figure 5, all E-cells receive the same constant drive, *I*_*E*_ = 2. This, of course, is not realistic. When the network is less perfect, for instance when different E-cells receive different amounts of drive, the suppression transition becomes broader. This is illustrated by Figure 6, which is analogous to 5, but with drive to the E-cells uniformly distributed between 1.85 and 2.15: The *i*-the E-cell receives drive

$$I_{E,i}=1.85+\frac{i-1/2}{160}\times 0.30,\;1\leq i\leq 160.$$

Panels **(A)** through **(C)** of Figure 6 show a transition regime in which stronger and weaker E-cell population spike volleys alternate somewhat erratically. Panel **(D)** of Figure 6 shows results of a simulation in which the I-cells were gap-junctionally coupled Erisir neurons. In this case, the transition regime is much narrower. It is also more orderly: the number of suppressed E-cells increases monotonically with *I*_*I*_. We discuss these two points in greater detail in sections 3.2 and 3.3.

### 3.2. With gap-junctions, I-cells of type 2 produce a tighter suppression transition than I-cells of type 1

Our main goal in this section is to explain why the suppression transition is gradual in Figure 5C, but sudden in Figure 5D. In these simulations, the E-cell population is tightly synchronous because there is no heterogeneity in drive to the E-cells, and the I-cell population is tightly synchronous because of gap-junctional coupling. Much about these simulations can therefore be understood by thinking about networks consisting of just one E-cell and one I-cell.

If one couples an RTM neuron with a WB neuron, there is typically a fairly broad range of drives, *I*_*I*_, to the I-cell for which the E-cells skip every second cycle; see Figure 7A for an example. Figure 7B shows the inter-spike intervals of the I-cell in this example: they alternate between longer and shorter intervals. On one cycle of the I-cell, the E-cell fires immediately prior to the I-cell. Because the excitatory synaptic currents have a positive decay time constant, the excitation resulting from the E-cell spike lingers slightly beyond the I-cell spike and into the next I-cell cycle. Recall that the WB neuron has a type 1 phase response curve; see Figure 3. The next I-cell spike is thereby advanced, and this can result in suppression of the E-cell on the next cycle.

Figures 7C,D show a similar experiment for a two-cell network consisting of an RTM neuron and an Erisir neuron. For the value of *I*_*I*_ = 7.27 shown in the figure, there is no cycle-skipping, and the network settles into an oscillation with a period of approximately 26 ms. If *I*_*I*_ is raised from 7.27 to 7.28 (not shown in the figure), the E-cell is suppressed altogether, and the period of the I-cell falls to approximately 23 ms. Because the I-cell is of type 2, the input from the E-cell, by lasting for a few milliseconds beyond the spike of the I-cell, does not advance the next I-cell spike; it delays it. Thus if the I-cell is unable to suppress the E-cell on a given cycle, it will be *delayed* on the next cycle, and is therefore even less able to suppress the E-cell on the next cycle. As a result, the E-cell is either suppressed on all cycles, or on none.

In Figure 8, we show further results of simulations for two-cell networks, illustrating the transition from gamma frequency firing to suppression of the E-cell, and in particular the effect of varying the strength of inhibition. Here we plot the number, *f*_*E*_, of E-cell spikes in 1000 ms (that is, the E-cell frequency in Hz) as a function of *I*_*I*_, with *g*_*IE*_ = 0.5 (blue), 0.8 (black), and 1.1 (red). The figure confirms that there is an abrupt suppression transition when the I-cell is an Erisir neuron (panel **B**), but not when it is a WB neuron (panel **A**).

In Figure 8, note that an increase in *I*_*I*_ typically causes either an abrupt drop in *f*_*E*_, or a slight increase. The reason for the increase in *f*_*E*_ with increasing *I*_*I*_ is that the enhanced drive to the I-cells makes them fire earlier on each cycle, thereby allowing the E-cell to fire earlier on the next cycle, unless there is a change in entrainment pattern, and the E-cell is suppressed on more cycles than before. When there is a change in entrainment pattern as a result of an increase in *I*_*I*_, the frequency *f*_*E*_ drops abruptly.

### 3.3. With gap-junctionally coupled I-cells of type 2, the dynamics in the transition regime are more regular than with I-cells of type 1

**Figure 9:**
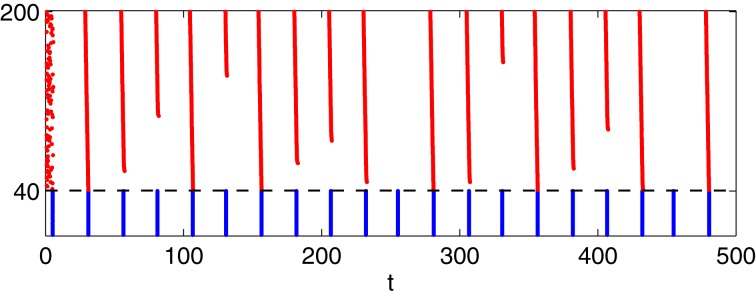
**Simulation with *I*_*I*_ = 0.9 fixed, all else as in panel (C) of Figure 6: I-cells were WB-neurons, coupled with gap junctions.** Drive to the E-cells was heterogeneous, increasing from 1.85 to 2.15 with increasing neuronal index.

There are seemingly irregular sequences of strong and weak E-cell spike volleys in Figure 6C, while much more regular behavior is seen in Figure 6D. We now discuss this difference. The behavior in Figure 6C becomes clearer when one fixes the mean drive, *I*_*I*_, to the I-cells in the transition regime. For illustration, Figure 9 shows a simulation similar to that of panel **(C)** of Figure 6—WB interneurons, gap junctions, heterogeneous drive to the E-cells—but with a fixed drive of 0.9 to the I-cells. We see a rather irregular sequence of stronger and weaker E-cell population spike volleys.

**Figure 10:**
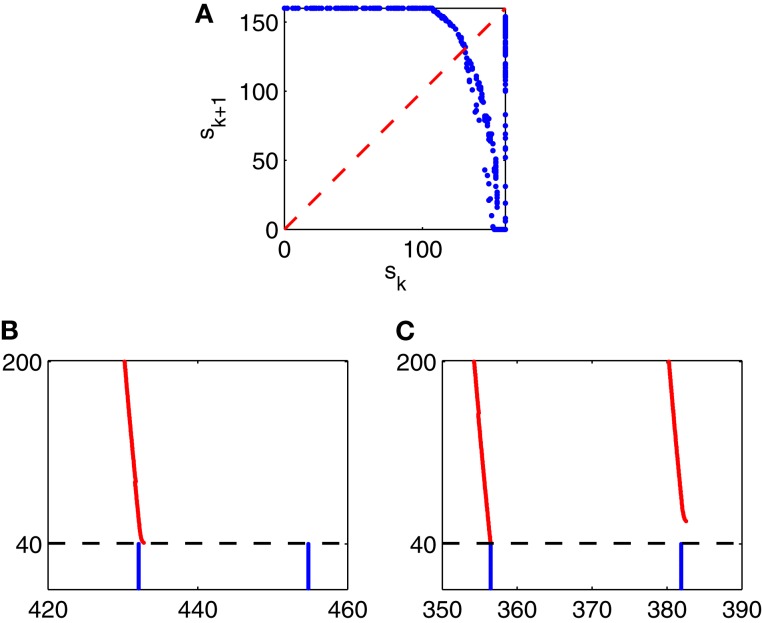
**(A)** The strength, *s*_*k* + 1_, of the (*k* + 1)-st spike volley as a function of the strength, *s*_*k*_, of the *k*-th, for the simulation of Figure 9, run over 10,000 ms. (**B** and **C**): Closeups of Figure 9: What happens after a full E-cell spike volley depends sensitively on when, relative to the E-cell spike volley, the I-cell spike volley occurs.

To understand this irregularity, we reduce the network dynamics to a one-dimensional map, examining how the strength of a given E-cell spike volley depends on the strength of the previous volley. We denote by *s*_*k*_ the number of E-cells that fire on the *k*-th gamma cycle in Figure 9. In Figure 10A, we have plotted the pairs (*s*_*k*_, *s*_*k* + 1_) for the simulation of Figure 9, run over 10,000 ms. If *s*_*k*_ < 160, then *s*_*k* + 1_ can be deduced from *s*_*k*_, approximately at least:

$$s_{k+1}=g(s_{k}),$$

and the figure suggests that *g* has exactly one fixed point s^*^, which is unstable: *g*′(x^*^) < −1. If *s*_*k*_ = 160, then *s*_*k* + 1_ is not determined by *s*_*k*_. Instead, *s*_*k* + 1_ then depends on the precise placement of the inhibitory spike volley in the *k*-th cycle, relatively to the excitatory one. If the inhibitory spike volley comes early, then the following I-cell spike volley comes so early that it largely or completely suppresses the E-cell spike volley; see Figure 10B. If the inhibitory spike volley comes late, then the following I-cell spike volley comes so late that it is ineffective at suppressing the E-cell spike volley on the next cycle; see Figure 10C.

To construct a figure analogous to Figure 10A for Erisir interneurons is more difficult. There is no transition regime in which the behavior is irregular, and therefore simply running a long simulation does not produce many pairs (*s*_*k*_, *s*_*k* + 1_). However, we describe now a method for constructing an idealized version of Figure 10A, and this method generalizes to the case of Erisir interneurons, leading to a better understanding of the difference between Figures 6C and 6D.

We start the simulation of the network, at time *t* = 0, in a point, *X*_0_, in phase space chosen so that a full population spike volley of the E-cells is imminent within a few milliseconds. Exactly how *X*_0_ is defined is largely irrelevant. We obtain it from the simulation of Figure 9 by recording all dependent variables 4 ms prior to the 5th population spike volley of the E-cells. (All E-cells happen to participate in that volley.) Within a few milliseconds, at time *t* = *t*_0_ > 0, we re-set the phase space variables associated with the I-cell population to a point *Y*_0_ to force an immediate population spike volley of the I-cells. Again, it is largely irrelevant exactly how *Y*_0_ is defined. We obtain it from the simulation of Figure 9 by recording all dependent variables associated with the I-cells at the onset of the 4th population spike volley of the I-cells.

**Figure 11:**
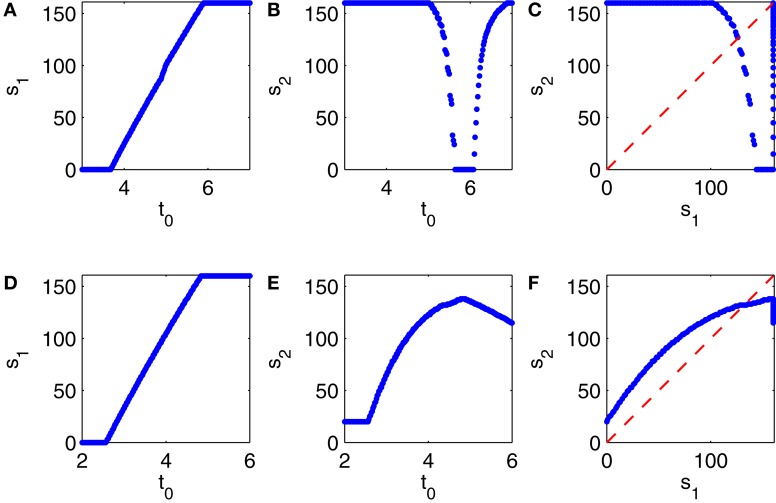
**(A–C)** Results from simulations of a PING network consisting of 160 RTM cells (E-cells) and 40 WB cells (I-cells). At the start of the simulation, at time 0, the E- and I-cells are close to firing spike volleys. At time *t*_0_ > 0, a spike volley of the I-cells is forced. **(A)** Strength of the first E-cell spike volley, *s*_1_, as a function of *t*_0_. **(B)** Strength of the second E-cell spike volley, *s*_2_, as a function of *t*_0_. **(C)** *s*_2_ as a function of *s*_1_. **(D–F)** Similar results for a network in which the I-cells are Erisir neurons.

Depending on the choice of *t*_0_, the first E-cell spike volley may be suppressed partially or completely. The number, *s*_1_, of E-cells firing soon after time *t* = 0, 0 ≤ *s*_1_ ≤ 160, is a function of *t*_0_ ≥ 0; see Figure 11A. We then record the number, *s*_2_, of E-cells firing in the second E-cell spike volley, and plot it as a function of *t*_0_ (Figure 11B), and also *s*_2_ as a function of *s*_1_ (Figure 11C). Note that Figure 11C is strikingly similar to Figure 10A.

The procedure used to generate Figures 11A–C can be applied to generate analogous figures for a network in which the I-cells are Erisir interneurons; see Figures 11D–F. The mean external drive to the I-cells in Figures 11D–F is *I*_*I*_ = 7 (this is near the lower end of the transition regime, see Figure 6D). Since the simulation with *I*_*I*_ = 7 does not produce any full E-cell population spike volleys, it cannot be used to initialize the network so that a full E-cell population spike volley is imminent; we therefore obtain the points *X*_0_ and *Y*_0_ just as described earlier, but based on a preliminary calculation with the reduced value *I*_*I*_ = 6.5, for which the E-cells do fire full spike volleys.

Comparing Figures 11C,F, we see that *s*_2_ is a decreasing function of *s*_1_ when the I-cells are WB neurons, but an increasing function of *s*_1_ when the I-cells are Erisir neurons. The difference in monotonicity is the crucial point here. It is a reflection of the difference in the types of the phase response curves: for type 1 interneurons, the firing of a greater number of E-cells accelerates the inhibitory response, reducing the number of E-cells firing on the next cycle; for type 2 interneurons, the opposite is true.

An iteration of the form *s*_*k* + 1_ = *g*(*s*_*k*_), where *g* = *g*(*s*) is a smooth function defined for 0 ≤ *s* ≤ 160 with 0 ≤ *g*(*s*) ≤ 160, may not have a stable fixed point when *g* is decreasing, but it does have at least one stable fixed point when *g* is increasing. So the mapping from *s*_1_ to *s*_2_ has an attracting fixed point when the interneurons are of type 2, but not necessarily, and in particular not in the example shown in Figure 11C, when they are of type 1. This is why type 2 interneurons yield periodic behavior (E-cell spike volleys of a steady size) after a transient, whereas type 1 interneurons may produce more complicated, possibly chaotic dynamics in the transition regime.

### 3.4. Networks without gap-junctional coupling among the I-cells

Simulations similar to that of Figure 5A were studied, for networks of theta neurons, in Börgers and Kopell (2005). Recall that the drive to the I-cells is heterogeneous; *I*_*I*_ denotes the mean drive to the I-cells. With increasing *I*_*I*_, increasingly many I-cells have enough drive to fire without being prompted by the E-cells. Once enough I-cells are in this group, the E-cells are suppressed altogether. In a network of cells coupled by inhibitory chemical synapses, but not by gap junctions, heterogeneity of drives typically prevents synchronization (White et al., 1998); this is why the I-cells in Figure 5A become asynchronous as soon as they begin firing without being prompted by E-cell spike volleys.

This discussion suggests that even in Figure 5A, considering that different I-cells receive different drives, the transition from PING to suppression of the E-cells should not be abrupt. Rather, one would expect that in an intermediate regime, some I-cells (the more strongly driven ones) are asynchronously active without being prompted by the E-cells, while others (the less strongly driven ones) only fire in synchronous volleys immediately following E-cell spike volleys. This is in fact so, but not visible in Figure 5A because the intermediate regime is quite narrow, and the rising *I*_*I*_ passes through it rapidly.

Figure 5B is in some regards similar to Figure 5A. The transition regime in which some I-cells fire without being prompted by the E-cells, but the E-cells still fire population spike volleys occasionally, is now much broader, and clearly visible. The most striking difference between Figures 5A,B is that in Figure 5B, some I-cells are completely suppressed even when the mean drive to the I-cells gets strong, whereas the same is not true in Figure 5A. We have not attempted a theoretical explanation of this difference; it seems natural to hypothesize that it is due to the difference in the types of the *f* - *I* relations of WB and Erisir neurons.

Figures 6A,B are similar. The suppression transitions are softened considerably because of heterogeneity in drive to the E-cells. With Erisir interneurons, one sees a *broader* suppression transition than with WB interneurons. This is opposite to what is seen in the presence of gap junctions. We have no explanation of this effect. In particular, our arguments based on the phase response of the I-cells do not apply when the I-cells are not kept synchronous by gap junctions: once the I-cells de-synchronize, they receive excitatory input from the E-cells at all phases, not just at the early phases at which type 2 I-cells are delayed by such inputs.

## 4. Discussion

The fast-spiking inhibitory basket cells believed to be central in the formation of gamma rhythms are in fact gap-junctionally connected (Beierlein et al., 2000). There is direct experimental evidence that they have type 2 phase response curves (Tateno and Robinson, 2007, Figure 5). In addition, there are reports that they also have type 2 frequency-current relations, i.e., that their firing starts at a non-zero frequency (Beierlein et al., 2003; Tateno et al., 2004; Tateno and Robinson, 2006, 2009). Further, there are several papers documenting resonance properties of fast-spiking interneurons; e.g., (Pike et al., 2000). These are properties usually associated with a Hopf bifurcation, and a type 2 phase response curve. It therefore appears that the biologically most relevant case is that of gap-junctionally connected I-cells with a type 2 phase response curve—precisely the case that yields the narrowest and most orderly suppression transition.

In this paper, we have discussed the loss of gamma rhythms due to too much excitation of the I-cells. By contrast, a recent paper of Börgers et al. (2012) investigated the loss of gamma rhythms due to too little excitation of the I-cells. Moca et al. (2012) discussed a different way in which the type of the interneurons may be important in gamma oscillations: they found that resonance properties of the interneurons, associated with bifurcation type 2, may contribute to stabilizing the gamma frequency.

All numerical experiments in this paper have been for “strong PING,” that is, for PING oscillations in which participating E-cells fire at or near gamma frequency. By contrast, “weak PING” oscillations are noise-driven, and individual participating E-cells fire on a small, randomly selected fraction of cycles only. In Börgers et al. (2005), it was suggested that weak PING might be associated with general alertness or vigilance, while strong PING might be a model of a cell assembly in a state of actively processing a specific item. We would expect our main conclusion, that I-cells with type 2 phase response curves result in a tighter suppression transition, to hold for weak PING as well. The reason is simply that for type 2 I-cells, the spiking of a few E-cells promotes the spiking of other E-cells on the next cycle, whereas for type 1 I-cells, it may make it harder for other E-cells to fire on the next cycle.

Our discussion suggests that the competition among E-cells associated with PING oscillations [see for instance (Olufsen et al., 2003; Börgers et al., 2008)] is less fierce when the I-cells have a type 2 phase response. We think that this is true only in a narrow time window: an E-cell that lags behind others by just a millisecond or two can fire more easily when the I-cells are type 2 than when they are type 1, since the firing of the E-cells that are ahead delays the firing of the I-cells on the next gamma cycle. In other words, I-cells of type 2 may allow for less tightly synchronous PING assemblies. However, even with I-cells of type 2, a cell assembly can suppress a less strongly driven competitor if the difference in drive is just slightly greater.

In summary, we have found that in the presence of gap junctions, when the I-cells are of type 2, the suppression transition tends to be both narrower and more orderly than when the I-cells are of type 1. It is tempting to speculate that fast-spiking inhibitory basket cells might have evolved to have type 2 phase response curves precisely because that leads to clean suppression transitions, reducing the amount of modulation of local recurrent inhibition needed to turn gamma frequency cell assemblies off or on, an operation that seems likely to be crucial in brain function.

### Conflict of interest statement

The authors declare that the research was conducted in the absence of any commercial or financial relationships that could be construed as a potential conflict of interest.

## Footnotes

^1^Two additional unstable fixed points come into existence as *I* rises above a critical value very close to 6.3, but that bifurcation is not our interest here, and is not shown in Figure 2.
